# Pennsylvania Hospital

**Published:** 1849-02

**Authors:** Spenser Sergeant

**Affiliations:** Resident Surgeon; Pennsylvania Hospital


PENNSYLVANIA HOSPITAL.—*Surgical Wards.*—*Service of* DR. NORRIS.

*Cases admitted since December 15th, 1848.*

Abscess 4, burns and scalds 4, cataract 1; fractures, simple, 6: viz.: leg 2, arm 2, fore arm 1, hand 1; compound 6, viz.: leg 1, hand 1, foot 1, skull 2, upper and lower maxillæ 1; frosted feet 1, gonorrhœa 2, hernia (reducible) 1, hydrocele 1, inflamed hand 2, inflamed testicle 1, paronychia 1, phymosis 2; syphilis, primary, 4, secondary 2; ulcers 7, varicose veins 2; wounds 13, viz.: contused 6, incised (of abdomen) 1, gun shot 1, lacerated 3, punctured 1. Total 59.

*Discharged since December 15th, 1848.*

| | Cured. | Relieved. | Died |
|---|---|---|---|
| Aneurism, (inguinal) - - - - | 1 | 0 | 0 |
| Balanitis, - - - - - - - - - | 2 | 0 | 0 |
| Burns and scalds, - - - - - | 2 | 0 | 2 |
| Chron. conjunctivitis, - - - - | 0 | 1 | 0 |
| Diseased foot, - - - - - - - | 1 | 0 | 0 |
| Fractures, simple— | | | |
| Thigh, - - - - - - - - - - - | 1 | 0 | 0 |
| Leg, - - - - - - - - - - - - | 1 | 0 | 0 |
| Patella, - - - - - - - - - - | 1 | 0 | 0 |
| Fore arm, - - - - - - - - - | 5 | 0 | 0 |

| | Cured. | Relieved. | Died. |
|---|---|---|---|
| Fractures, compound— | | | |
| Skull, - - - - - - - - - | 0 | 0 | 1 |
| Toes, - - - . - - - - - | 1 | 0 | 0 |
| Foot, - - - - - - - - - | 0 | 0 | 1* |
| Gonorrhœa, - - - - - - - | 1 | 0 | 0 |
| Hemorrhoids - - - - - - - | 2 | 0 | 0 |
| Inflamed hand, - - - - - - | 2 | 0 | 0 |
| Do. leg, - - - - - - - | 1 | 0 | 0 |
| Do. scrotum, - - - - - | 1 | 0 | 0 |
| Necrosis (of head,) - - - - - | 1 | 0 | 0 |
| Syphilis, - - - - - - - - | 3 | 0 | 0 |
| Sprain, - - - - - - - - | 1 | 0 | 0 |
| Stricture of the urethra, - - | 0 | 0 | 1† |
| Tumor, - - - - - - - - | 2 | 0 | 0 |
| Wounds, viz.: contused 6 ; incised 4 ; lacerated 2, - - | 12 | 0 | 0 |
| Ulcers, - - - - - - - - | 5 | 0 | 0 |
| Total, | 46 | 1 | 5 |

SPENSER SERGEANT, M. D.,
*Resident Surgeon.*

*Pennsylvania Hospital*, January 15th, 1845.

* An intemperate man ; his foot was dreadfully crushed by a rail road car. Amputation was performed soon after reaction, but he died on the fifth day, of gangrene of the stump.

† Stricture of several years standing. Treatment by dilatation adopted. Died of disease of kidneys.

